# Biofabrication for complex tissue regeneration: a scoping review of translational gaps in craniomaxillofacial reconstruction

**DOI:** 10.3389/froh.2026.1812860

**Published:** 2026-05-28

**Authors:** Shantanu Dixit, Goma Kathayat, Dinesh Rokaya, Ahmad Al Jaghsi, Suphachai Suphangul

**Affiliations:** 1 Dental Imaging and Diagnostic Center, Ballabgarh, Haryana, India; 2 Chulalongkorn University, Bangkok, Thailand; 3 College of Dentistry, Ajman University, Ajman, United Arab Emirates; 4 Mahidol University—Phayathai Campus, Bangkok, Thailand

**Keywords:** biofabrication, biomaterials, craniomaxillofacial, tissue engineering, translational research

## Abstract

**Introduction:**

Reconstruction of the craniomaxillofacial (CMF) region poses combined structural and biological challenges that are difficult to address using conventional approaches. Although biofabrication has gained attention in this context, its progression across fabrication strategies and stages of translational validation has not been examined in a consolidated manner.

**Methods:**

A scoping review was conducted in accordance with PRISMA-ScR recommendations. Relevant studies published between 2010 and 2025 were identified and examined using a four-dimensional analytical framework (spatial, biological, temporal, and biomechanical complexity) to evaluate fabrication strategy, construct complexity, bio-additive integration, and translational maturity.

**Results:**

Analysis of 107 studies indicated that research activity clustered around a limited set of approaches, most frequently extrusion-based printing (70.1%) in combination with composite material systems (59.8%). Although 21.5% of studies investigated complex multi-tissue constructs, most of these were evaluated exclusively in vitro or in small animal models. Bio-additive incorporation predominantly focused on osteogenic enhancement, with relatively limited attention to angiogenic support. Across studies, outcome reporting favored structural characteristics over assessments of functional recovery.

**Discussion:**

These findings highlight a gap between increasing construct complexity in CMF biofabrication and validation in functionally and clinically relevant models. Progress toward translation is likely to depend on closer integration of fabrication strategies, biological design considerations, and function-oriented evaluation frameworks.

**Systematic Review Registration:**

Doi: 10.17605/OSF.IO/FJ87Y.

## GRAPHICAL ABSTRACT

**Figure:**
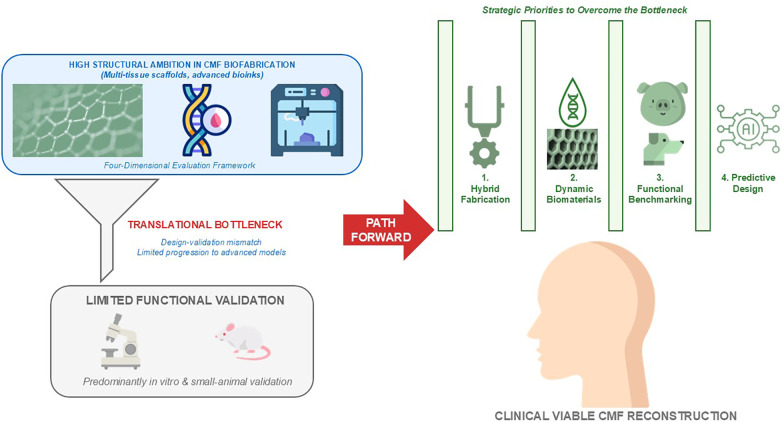
Translational Ambition Gap and Strategic Roadmap for Biofabrication. Using the craniomaxillofacial region as a stress-test model, this conceptual framework synthesizes the core finding of this review: a systemic disconnect between increasing construct complexity (left) and the rigor of functional/clinical validation (right). To bridge this gap, four integrated strategic pillars are proposed: Hybrid Fabrication, Dynamic Biomaterials, Functional Benchmarking, and Predictive Design. The co-development of agile regulatory pathways forms the essential foundation for translating this roadmap into clinical reality

## 1 Introduction

The craniomaxillofacial (CMF) region is susceptible to a wide spectrum of conditions ranging from trauma, surgical defects, and congenital anomalies to relatively common dentoalveolar diseases like periodontitis or dental caries (1, 2). These conditions manifest in load-bearing regions like calvaria, mandible, or dentition, as well as delicate regions like the mid-facial skeleton. Overall, their sequelae result in numerous morbidities varying from compromised esthetics to functional impairments in articulation, mastication, or swallowing, ultimately deteriorating an individual's quality of life (3).

CMF reconstruction is challenging due to its complex three-dimensional anatomy, complicated vasculature, and conventional graft limitations (4). This challenge is exemplified by defects such as those in the calvarium or alveolus, where regeneration demands the precise arrangement of multiple tissues. A more complex case is periodontitis, which requires the simultaneous restoration of cementum, periodontal ligament, and alveolar bone (1, 5).

While conventional grafting via autografts, allografts, or xenogeneic grafts has long been a benchmark for defect repair in the CMF region (2), its efficacy is limited by several disadvantages, such as donor site morbidity, limited availability, immunogenicity risk, unpredictable integration, and limited biointegration (6). To overcome these limitations, additive manufacturing, commonly known as three-dimensional (3D) printing or rapid prototyping, was introduced (7). This technology utilizes fabrication methods like FDM (fused deposition modeling), DLP (digital light processing), SLS (selective laser sintering), or SLA (stereolithography) (2, 5). This enabled the production of patient- and site-specific, customizable metal or bioceramic-based acellular scaffolds using CAD/CAM software (8). However, these acellular scaffolds, while anatomically precise, offered limited bioactivity. This critical deficit—the ability to replicate geometry but not biology—directly spurred the development of biofabrication (2, 5).

The limitations of 3D-printed scaffolds, combined with the principles of tissue engineering, gave rise to the concept of biofabrication (2, 9). This field addresses the bio-inertness of the former approach through the automated generation of biologically functional constructs with or without scaffolds. It achieves this by incorporating living cells and bioactive molecules into fabrication processes, utilizing techniques such as bioprinting and bioassembly. Numerous reviews (1, 2, 5, 10) have reinforced the existing literature with an intensified focus on 3D bioprinting, including our own prior work (10). In this context, the present review differs in several key aspects: it encompasses the full spectrum of biofabrication strategies (scaffold-assisted, cell-laden, hybrid, and cell-free); introduces a conceptual four-dimensional analytical framework to assess translational readiness across spatial, biological, temporal, and biomechanical dimensions; provides a comprehensive synthesis of 107 studies with structured gap analysis; and proposes an actionable strategic roadmap for clinical translation. Biofabrication in the CMF region poses several challenges. Along with mechanical support, it also requires functional integration across a heterogeneous set of tissues. The periodontal ligament complex clearly illustrates this regenerative challenge (1, 5). Vascularization is essential to promote biological and functional integration between constructs and host tissues (4, 8). The necessity of meeting these mechanical, multi-tissue, and vascular integration demands makes the CMF region a unique stress-test environment for biofabrication strategies. This review examines CMF reconstruction as a model system to assess current biofabrication strategies and identify potential barriers to clinical translation (4, 5). Insights from this region may also inform approaches in other areas of complex tissue engineering.

## 2 Aims and objectives

This scoping review aims to outline the extent of application, progress, and translational hurdles of biofabrication, including bioprinting, in CMF reconstruction. Guided by the Population–Concept–Context (PCC) framework, our specific objectives are: a) Population: Assessment of invitro/*in vivo* models, cell sources, and target tissues related to CMF reconstruction; b) Concept: Map the employed biofabrication strategy, material systems, etc.; c) Context: Synthesize the translational progress and identify the key hurdles. This review was designed to answer the following research question: “What is the extent, range, and nature of existing evidence on biofabrication strategies for CMF reconstruction?”.

## 3 Materials and methods

### 3.1 Protocol and registration

We conducted this review following the JBI methodology and reported using PRISMA-ScR guidelines (11, 12). Before commencing the review, we drafted and publicly registered a study protocol with the Open Science Framework (OSF) on 29 August 2025. The original registration was accidentally withdrawn after manuscript acceptance; the protocol was re-registered on 8 May 2026 and is available at https://osf.io/2e8rz. (DOI: 10.17605/OSF.IO/HN5PQ).

### 3.2 Selection criteria

Study eligibility was determined according to the predefined PCC framework, with the following operational criteria:

Inclusion Criteria: Primary research article (i) published in English between January 2010 and June 2025; (ii) employ fabrication technology like biofabrication, additive manufacturing, or 3D bioprinting for tissue regeneration related to the CMF region; (iii) involve the biological models (*in vitro* or *in vivo*) and/or reporting on cell sources relevant to CMF tissues; (iv) focus on CMF applications like calvarium, alveolar bone, maxillofacial bone, tooth, periodontium, temporomandibular joint (TMJ), and composite tissue defects; and (v) report the key outcomes relevant to tissue regeneration.

Exclusion criteria: We excluded review articles and meta-analyses from the formal analysis, though we did use them to provide context in the narrative sections of this review. Studies focusing on engineering aspects, non-CMF applications, and non-English publications were also excluded.

### 3.3 Information sources and search strategy

Searches were conducted on June 15, 2025. A comprehensive search was performed across two primary bibliographic databases, PubMed and Scopus, which were selected for comprehensive biomedical coverage. This was supplemented by a targeted search of Google Scholar (first 300 results) to identify additional grey literature and conference proceedings not indexed in major databases. The restriction to 300 results was based on relevance ranking and practical feasibility, as screening beyond this threshold in preliminary testing yielded no additional eligible studies. Search terms included both “biofabrication” and “3D bioprinting” to reflect differences in terminology used across studies and to maximize retrieval of relevant literature. Database search strings are detailed in Table 1. The search covered articles published between January 2010 and June 2025.

**Table 1:** Systematic search strategy used for the identification of studies on biofabrication strategies for CMF reconstruction.

Database	Search String
PubMed	(“biofabrication”[Title/Abstract] OR “3D bioprinting”[Title/Abstract] OR “three-dimensional bioprinting”[Title/Abstract] OR “bioprint*”[Title/Abstract] OR “additive manufacturing”[Title/Abstract])
	AND
	(“oral and maxillofacial”[Title/Abstract] OR craniofacial[Title/Abstract] OR mandible[Title/Abstract] OR maxilla[Title/Abstract] OR “alveolar bone”[Title/Abstract] OR periodontal[Title/Abstract] OR “dentin-pulp complex”[Title/Abstract] OR “dental pulp”[Title/Abstract] OR TMJ[Title/Abstract] OR “temporomandibular joint”[Title/Abstract] OR gingiva[Title/Abstract] OR “oral mucosa”[Title/Abstract])
	AND
	(reconstruction[Title/Abstract] OR regeneration[Title/Abstract] OR “tissue engineering”[Title/Abstract] OR scaffold[Title/Abstract] OR bioink[Title/Abstract] OR hydrogel[Title/Abstract] OR biomaterial*[Title/Abstract])
Scopus	(TITLE-ABS-KEY(“biofabrication”) OR TITLE-ABS-KEY(“3D bioprinting”) OR TITLE-ABS-KEY(“three-dimensional bioprinting”) OR TITLE-ABS-KEY(bioprint*) OR TITLE-ABS-KEY(“additive manufacturing”))
	AND
	(TITLE-ABS-KEY(“oral and maxillofacial”) OR TITLE-ABS-KEY(craniofacial) OR TITLE-ABS-KEY(mandible) OR TITLE-ABS-KEY(maxilla) OR TITLE-ABS-KEY(“alveolar bone”) OR TITLE-ABS-KEY(periodontal) OR TITLE-ABS-KEY(“dentin-pulp complex”) OR TITLE-ABS-KEY(“dental pulp”) OR TITLE-ABS-KEY(tmj) OR TITLE-ABS-KEY(“temporomandibular joint”) OR TITLE-ABS-KEY(gingiva) OR TITLE-ABS-KEY(“oral mucosa”))
	AND
	(TITLE-ABS-KEY(reconstruction) OR TITLE-ABS-KEY(regeneration) OR TITLE-ABS-KEY(“tissue engineering”) OR TITLE-ABS-KEY(scaffold) OR TITLE-ABS-KEY(bioink) OR TITLE-ABS-KEY(hydrogel) OR TITLE-ABS-KEY(biomaterial*))
Google Scholar^†^	Search Formula: (“biofabrication” OR “3D bioprinting” OR “three-dimensional bioprinting” OR bioprinting OR “additive manufacturing”) AND (“oral and maxillofacial” OR craniofacial OR mandible OR maxilla OR “alveolar bone” OR periodontal OR “dentin-pulp complex” OR “dental pulp” OR TMJ OR “temporomandibular joint” OR gingiva OR “oral mucosa”) AND (reconstruction OR regeneration OR “tissue engineering” OR scaffold OR bioink OR hydrogel OR biomaterials)
	Conceptual Breakdown:
	Biofabrication Concept AND Anatomy Concept AND Regeneration Concept

† The first 300 results from Google Scholar, sorted by relevance, were screened.

The Google Scholar search is described using both the actual search formula attempted and a conceptual breakdown due to the platform's variable interpretation of Boolean operators.

### 3.4 Study selection process

**Figure 1:**
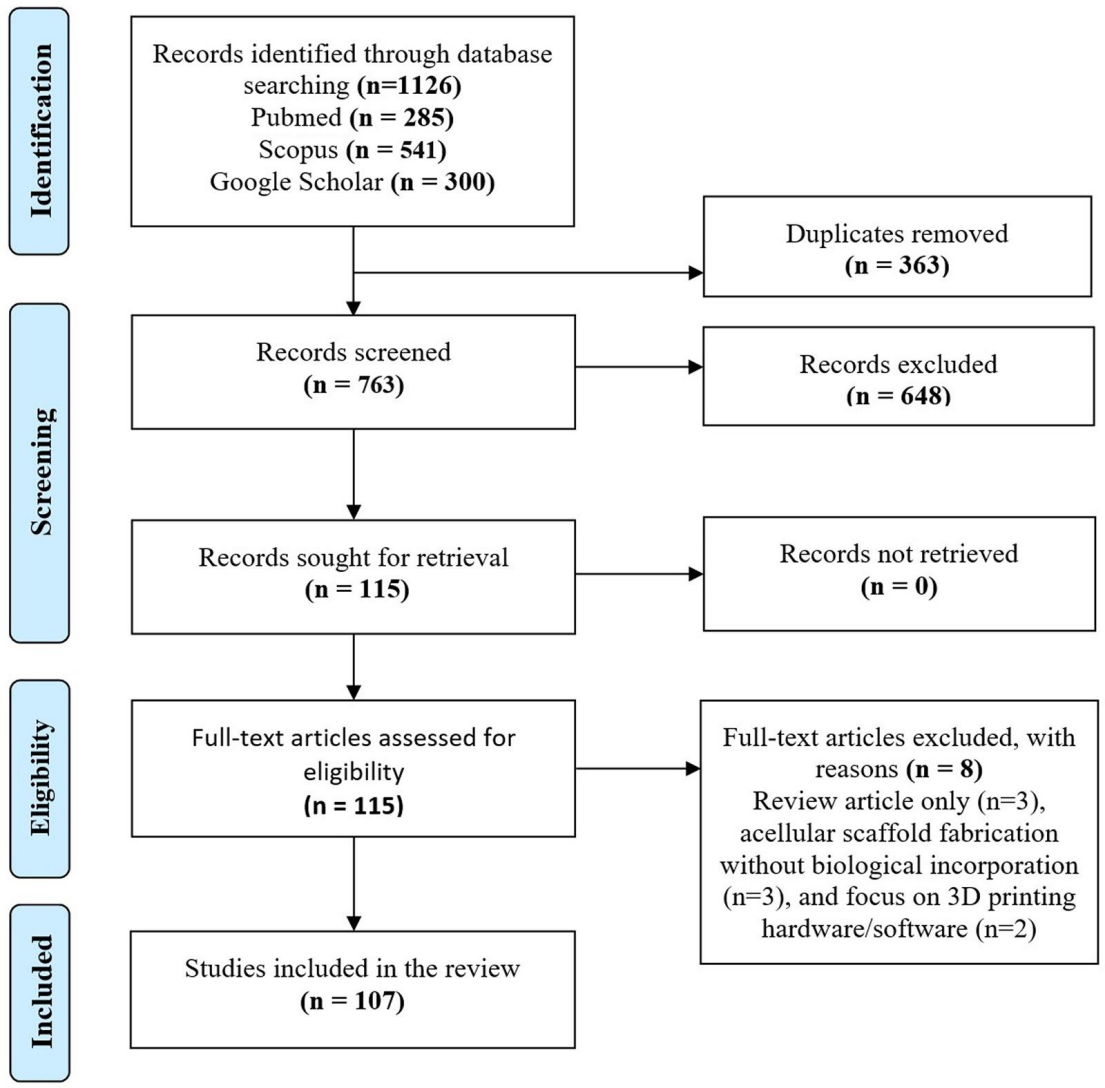
PRISMA flow diagram for systematic literature review.

All search records were managed using reference management software, followed by the removal of duplicate entries by both automated deduplication and manual verification. Two reviewers (S.D. and D.R.) then independently performed the title and abstract screening, followed by a full-text review. Reviewers used the following operational definitions for the screening: (a) CMF-Relevance via Cell Source: We included studies that used biologically relevant CMF cell sources (e.g., DPSCs, PDLSCs, SCAP), even if the experimental model itself was not CMF-specific. (b) CMF-Relevance via Anatomical Context: For studies using generic cell types (e.g., BM-MSCs), we required an explicit statement of a CMF application (e.g., mandible, maxilla, TMJ) in the study's aims or methods. The whole process of selection and study inclusion/exclusion is summarised in Figure 1. Disagreements between reviewers were resolved through discussion; if consensus could not be reached, a third reviewer (G.K.) was consulted. No formal agreement assessment was performed, as screening decisions were straightforward based on predefined eligibility criteria.

### 3.5 Classification framework

To ease the process of study segregation, according to biofabrication strategy, translational progression, engineering complexity, and cell sources, we developed and applied the following classification frameworks.

#### 3.5.1 Biofabrication strategy

Studies were categorised into four categories: scaffold-assisted (acellular scaffold with post-print cell seeding), cell-laden (scaffold with encapsulated cells), hybrid (combination of the former two), and cell-free (bioactive component rather than cells).

#### 3.5.2 Translational progression

Studies were divided into a four-tier system based on research translational maturity: *in vitro* (cell culture model), preclinical (small animals like rodent or rabbit model), preclinical (large animals like pigs, sheep, or dogs), and proof-of-concept/*ex vivo* (human tissue).

#### 3.5.3 Engineering complexity

Constructs were categorized according to increasing architectural and biological complexity using a four-level framework. Level I (mono-tissue) included constructs intended to regenerate a single tissue type, such as bone or dental pulp. Level II (bi-phasic) comprised constructs designed for two integrated tissues or for a single tissue-interface construct, such as the periodontal ligament (PDL) or cementum, where successful regeneration inherently depends on the formation of two opposing attachment interfaces. Level III (multi-tissue) encompassed constructs targeting functional junctions between three or more tissues, as well as combinations of multiple adjacent tissues, exemplified by the complete periodontal complex or composite craniofacial constructs. Level IV (whole organ or sector) referred to constructs aiming to replace an entire anatomical organ or a major functional portion thereof, such as a whole tooth.

#### 3.5.4 Cell sources

Studies were also divided based upon the cell sources in the following categories such as mesenchymal and non-mesenchymal cells from oral and other sources, cell lines, co-cultures, host-derived/host recruitment, and acellular.

### 3.6 Data extraction and charting

Following the PRISMA-ScR guidelines, two reviewers independently extracted the data into a standardized form in Microsoft Excel. The data extraction form captured the following variables for each study: author(s), year, country, target tissue, anatomical site, engineering complexity level (I–IV), translational phase, bioprinting strategy, cell source, fabrication technique, construct composition, biomaterial class, and key outcomes.

## 4 Results

### 4.1 Study selection and publication trends

A total of 1,126 records were identified through database searches. After removing duplicates and screening titles and abstracts, 115 full-text articles were assessed for eligibility, of which 107 met the inclusion criteria. These studies were published between 2010 and 2025, with 71 studies (66.3%) appearing in the last five years, indicating a recent surge in research activity. Study characteristics, including detailed methodology and outcomes, are presented in Supplementary Table S1.

### 4.2 Geographical distribution and translational stage

The included studies originated from 19 countries. China contributed the largest number (*n* = 36), followed by the United States (*n* = 21) and South Korea (*n* = 14), together accounting for two-thirds of all studies. Additional contributions came from Taiwan (*n* = 5), Australia (*n* = 4), France (*n* = 4), and Turkey (*n* = 4), with the remaining 19 studies distributed across 12 other countries (Table 2).

**Table 2:** Country-wise distribution of included studies stratified by translational stage.

Country	*In vitro*	Preclinical (Small animal)	Preclinical (Large animal)	Proof-of-concept	Number of studies
China	7	19	10	0	36
USA	6	13	2	0	21
South Korea	6	5	3	0	14
Taiwan	3	1	1	0	5
Australia	2	2	0	0	4
France	1	3	0	0	4
Turkey	3	1	0	0	4
Germany	1	1	0	1	3
Italy	2	0	1	0	3
Canada	1	1	0	0	2
Iran	1	1	0	0	2
Netherlands	2	0	0	0	2
Belgium	1	0	0	0	1
Brazil	1	0	0	0	1
Colombia	1	0	0	0	1
Japan	1	0	0	0	1
Portugal	1	0	0	0	1
Singapore	1	0	0	0	1
UK	1	0	0	0	1
**Grand Total**	**42**	**47**	**17**	**1**	**107**

Bold values indicate the total number of studies per country.

Studies were also classified by translational stage. *In vitro* models accounted for 42 studies (39.3%), small animal models for 47 studies (43.9%), and large animal models for 17 studies (15.9%). One *ex vivo* proof-of-concept study (0.9%), involving human tissue, was identified from Germany. Notably, countries contributing the highest number of studies also accounted for the majority of large-animal investigations, indicating a concentration of advanced preclinical work within a limited set of research ecosystems.

### 4.3 Engineering complexity

Studies were analysed using the four-level engineering complexity classification described in Section 3.5.3. Most included studies focused on mono-tissue constructs (Level I), which accounted for 71 studies (66.4%). Bi-phasic constructs (Level II) were less common, representing 11 studies (10.3%), and primarily involved two-tissue interfaces such as dentin–pulp or PDL-based junctions. Constructs addressing multi-tissue combinations and interfaces (Level III) were reported in 23 studies (21.5%). In contrast, whole-organ or sector constructs (Level IV) remained rare, with only two studies (1.9%). This distribution indicates that CMF biofabrication research remains predominantly focused on mono-tissue approaches, with progressively fewer studies tackling higher complexity challenges.

### 4.4 Target anatomical sites

Bone was the most frequently targeted tissue, appearing in 51 studies (47.7%). These comprised calvarial bone (*n* = 15), alveolar bone (*n* = 12), mandibular bone (*n* = 9), craniofacial bone without further regional specification (*n* = 6), and studies in which the bone site was not explicitly stated (*n* = 6). An additional three studies addressed other site-specific osseous targets (e.g., maxillary sinus or TMJ condyle) without fitting into the above anatomical categories.

Following bone, the periodontal complex was the second most investigated target tissue (18 studies, 16.8%), while multi-tissue constructs accounted for 11 studies (10.3%). Other tissues included dental pulp (*n* = 5), dentin–pulp complex (*n* = 5), gingiva (*n* = 5), cartilaginous tissues—predominantly the TMJ disc (*n* = 5), as well as dentin (*n* = 3), enamel (*n* = 1), tooth root (*n* = 1), and whole-tooth constructs (*n* = 1) (Table 3).

**Table 3:** Distribution of target tissues across translational stages in CMF biofabrication studies.

Target tissue/anatomical site	*In vitro*	Preclinical (Small animal model)	Preclinical (Large animal model)	Proof-of-concept (*ex vivo*)	Grand total	Representative studies
**Bone**	**14**	**30**	**7**	**0**	**51**	
Alveolar bone	4	5	3	0	12	(13–24)
Calvaria	1	14	0	0	15	(23, 25–38)
Mandible	1	6	2	0	9	(39–47)
Craniofacial	3	3	0	0	6	(48–53)
Not Specified	5	1	0	0	6	(54–59)
Others (Calvaria and Alveolar Bone, Maxillary Sinus, TMJ Condyle)	0	1	2	0	3	(60–62)
**Cartilage**	**3**	**2**	**1**	**0**	**6**	
Nasal/Auricular	0	0	1	0	1	(63)
TMJ Disc	3	2	0	0	5	(64–68)
**Dental Pulp**	**3**	**0**	**1**	**1**	**5**	(69–73)
**Dentin**	**1**	**2**	**0**	**0**	**3**	(74–76)
**Dentin-Pulp Complex**	**5**	**0**	**0**	**0**	**5**	(77–81)
**Enamel**	**1**	**0**	**0**	**0**	**1**	(82)
**Gingiva**	**2**	**2**	**1**	**0**	**5**	(83–87)
**Multi-Tissue Constructs**	**4**	**5**	**2**	**0**	**11**	(88–98)
**Periodontal Complex**	**9**	**6**	**3**	**0**	**18**	
Single-Component (Periodontal ligament; PDL)	2	2	1	0	5	(99–103)
Single-Component (Cementum)	1	0	0	0	1	(104)
Complex Periodontal Interfaces (e.g., Alveolar Bone-Cementum; Bone-PDL-Gingiva)	6	4	2	0	12	(105–116)
**Tooth Root**	**0**	**0**	**1**	**0**	**1**	(117)
**Whole Tooth**	**0**	**0**	**1**	**0**	**1**	(118)
**Grand Total**	**42**	**47**	**17**	**1**	**107**	

Bold values denote major tissue categories; indented entries are subtypes under each category.

### 4.5 Biomaterial, cell source, and bioprinting strategies

The selection of biomaterials and bioprinting strategies differed according to the anatomical site being targeted (Table 4). In bone regeneration studies, clear site-dependent patterns were evident. Mandibular bone constructs (*n* = 9) predominantly relied on scaffold-assisted approaches, with 8 studies adopting this strategy. A similar trend was observed for alveolar bone constructs, where scaffold-assisted methods were used in 9 of 12 studies. In contrast, calvarial bone studies (*n* = 15) demonstrated greater methodological heterogeneity, with cell-laden approaches most frequently reported (8 studies), followed by scaffold-assisted (5 studies) and hybrid strategies (2 studies).

**Table 4:** Summary of dominant biofabrication strategies by CMF tissue.

Target tissue/anatomical site	Study count	Dominant biomaterial strategy	Primary cell source	Dominant bioprinting strategy	Dominant fabrication technology
Bone (Alveolar Bone)	12	Composite Systems	Oral MSCs/Host derived/Other MSCs	Scaffold assisted	Extrusion
Bone (Calvaria)	15	Natural Polymers and Composites	Other MSCs/Oral MSCs/Co-culture Systems	Cell laden	Extrusion
Bone (Mandible)	9	Composite (Syn-Cer)	Host-derived/Other MSCs	Scaffold assisted	Extrusion
Bone (Craniofacial)	6	Composite (Nat-Cer)	Other MSCs	Cell laden	Extrusion
Other bone site (Maxillary Sinus, TMJ Condyle, Calvaria and Alveolar)	3	Varied (Ceramic, Composite)	Varied (Host-derived, Co-culture)	Scaffold assisted	Varied (Extrusion, SLS)
Bone (Site Not Specified)	6	Composite	Oral MSCs	Varied (Cell-laden, Scaffold-assisted)	Extrusion
Cartilage (TMJ Disc)	5	Synthetic Polymers and Composites	Other MSCs/Co-culture Systems	Scaffold assisted	Extrusion
Cartilage (Nasal/Auricular Cartilage)	1	Composite (Syn-Nat)	Chondrocytes	Scaffold assisted	SLS
Dental Pulp	5	Composite (Nat-Cer)	Oral MSCs	Cell laden	Extrusion
Dentin	3	Composite Systems	Oral MSCs	Cell laden	Extrusion
Dentin-Pulp Complex	5	Composite (Nat-Cer)	Oral MSCs	Cell laden/Hybrid	Extrusion
Enamel	1	Natural Polymers	Other Cells/Cell Lines	Cell laden	Extrusion
Gingiva	5	Natural polymer	Oral Cells (Non-Mesenchymal)	Cell laden	Extrusion
Multi-Tissue Constructs	11	Composite Systems	Co-culture Systems	Scaffold assisted	Extrusion
Periodontal Complex	18	Composite and Natural Polymers	Oral MSCs/Co-culture Systems	Cell laden	Extrusion
Tooth Root	1	Composite (Nat-Cer)	Oral MSCs	Scaffold assisted	Extrusion
Whole Tooth	1	Composite (Syn-Cer)	Oral MSCs	Scaffold assisted	Extrusion

Dominant trends were determined by descriptive frequency analysis of the charted data. Categorization follows predefined frameworks for cell sources (Section 3.5.3) and bioprinting strategy (Section 3.6).

MSCs, mesenchymal stromal cells; Nat-Cer, natural polymer-ceramic composite; Syn-Cer, synthetic polymer-ceramic composite; Syn-Nat, synthetic-natural polymer composite; SLS, selective laser sintering. Cell Source Categories (Section 3.5.3): Oral MSCs, dental/oral-derived stem cells (e.g., DPSCs, PDLSCs, SCAPs); Other MSCs, non-oral mesenchymal stem cells (e.g., BMSCs, ADSCs); Host-derived, *in situ* recruitment of host cells; Co-culture Systems, use of two or more distinct cell types; Oral Cells (Non-Mesenchymal), e.g., gingival fibroblasts, dental epithelial cells; Other Cells/Cell Lines, defined non-stem cell types or immortalized lines.

Dental and soft tissue constructs—including dental pulp, dentin–pulp complex, gingiva, and enamel (*n* = 16)—were fabricated almost exclusively using cell-laden approaches, reported in 15 studies. Periodontal complex constructs (*n* = 18) showed a more distributed use of strategies, with cell-laden constructs remaining the most common (9 studies), alongside scaffold-assisted (6 studies) and hybrid approaches (2 studies); one study employed a cell-free, vesicle-based method. Multi-tissue constructs (*n* = 11) similarly utilized a mix of scaffold-assisted (5 studies), cell-laden (4 studies), and hybrid strategies (2 studies), reflecting the technical demands of regenerating tissue interfaces.

**Figure 2:**
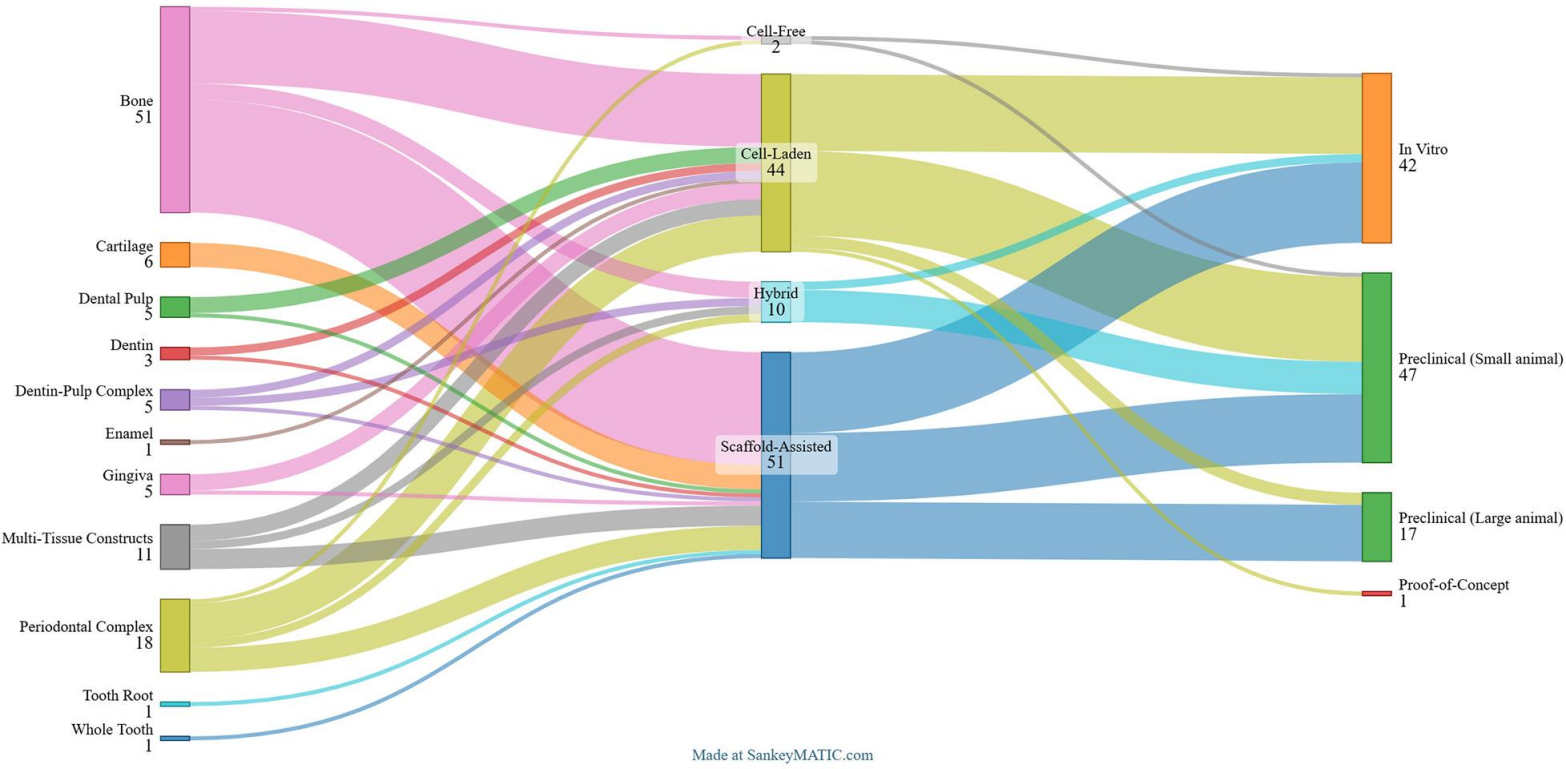
Systems-level mapping of biofabrication strategies and translational progress (craniomaxillofacial reconstruction). Sankey diagram of 107 studies showing flows from target tissue (left), through biofabrication strategy (center), to translational phase (right); line widths indicate study frequency. Most efforts focus on bone regeneration via scaffold-assisted (50) or cell-laden (43) approaches. Despite increasing construct complexity, a clear translational bottleneck emerges: 47 studies reached small-animal validation, 17 (15.9%) progressed to large-animal models, and a single study (0.9%) achieved proof-of-concept clinical translation. While derived from CMF applications, this pattern reflects broader tendencies in complex, load-bearing tissue biofabrication, highlighting systemic barriers between design sophistication and functional validation.

To integrate these tissue-specific strategy patterns within the broader translational context, we constructed a Sankey diagram (Figure 2), which maps the flow of all 107 studies across three analytical dimensions: target tissue, biofabrication strategy, and translational phase. The visualization confirms the patterns detailed above while providing additional integrative insights. Bone tissue dominates the inflow (51 studies), distributing primarily to scaffold-assisted (28 studies) and cell-laden (18 studies) approaches. The middle tier reveals the field's strategic balance, with scaffold-assisted (51 studies) and cell-laden (44 studies) as parallel dominant paradigms, consistent with the overall frequencies reported below.

Critically, the diagram visualizes a pronounced translational bottleneck. While 47 studies (43.9%) reached small animal validation, the flow narrows dramatically to only 17 studies (15.9%) at the large animal stage, with a single proof-of-concept study (0.9%) representing near-clinical work. This bottleneck persists across all strategy types, suggesting a field-wide challenge in advancing biofabricated constructs.

Across all studies, composite biomaterials represented the most frequently used material class (64 studies), followed by natural polymers (29 studies) (Table 5). Polycaprolactone (PCL, *n* = 30) and hydroxyapatite (HA, *n* = 29) were the most reported individual materials, often combined with collagen- or gelatin-based matrices. Scaffold-assisted (51 studies) and cell-laden strategies (44 studies) were employed at comparable overall frequencies, whereas hybrid (10 studies) and cell-free approaches (2 studies) were relatively uncommon. Extrusion-based bioprinting was the dominant fabrication technique (73 studies). When including hybrid strategies that utilized extrusion as a component method, the total reaches 75 studies (70.1%). Oral-derived mesenchymal stem cells were the most frequently reported cell source (37 studies).

**Table 5:** Biomaterial, cell, and technology landscape.

Category	Subgroup	Study count	Percentage
Biomaterial Class	Composite	64	59.80%
	Natural Polymers	29	27.10%
	Synthetic Polymers	9	8.40%
	Ceramics	5	4.70%
Prevalent Materials	Polycaprolactone (PCL)	30	28.00%
	Hydroxyapatite (HA)	29	27.10%
	Collagen	20	18.70%
	Gelatin Methacryloyl (GelMA)	19	17.80%
	Alginate	15	14.00%
	Gelatin	15	14.00%
	Tricalcium Phosphate (TCP)	14	13.10%
Cell Source	Oral MSCs/Dental Stem cells	37	34.60%
	Other MSCs	20	18.70%
	Co-culture Systems	19	17.80%
	Host-derived/Recruitment	11	10.30%
	Oral cells (Non-Mesenchymal)	6	5.60%
	Other cells (Non-Mesenchymal)/cell lines	6	5.60%
	Not specified/Not Applicable	6	5.60%
	Acellular (Vesicle-Based)	2	1.90%
Bioprinting Strategy	Scaffold-Assisted	51	47.70%
	Cell-Laden	44	41.10%
	Hybrid	10	9.30%
	Cell-Free	2	1.90%
Fabrication Technology	Extrusion-based	73	68.20%
	Inkjet-based	6	5.60%
	Vat Polymerization (Stereolithography/Digital Light Processing)	5	4.70%
	Other Technologies	23	21.50%
Multi-Technology Integration	Hybrid Fabrication Systems	5	4.70%

### 4.6 Bio-additive integration

Bio-additives were incorporated in 39 of the 107 included studies (36.4%) to direct biological processes such as osteoinduction, angiogenesis, and immunomodulation (Table 6), with usage varying by tissue target. Osteoinductive agents—including BMP-2/7, nanosilicates, and bioactive glass—were used in 16 studies (15.0%). In contrast, angiogenic factors, primarily VEGF, appeared in only 1 study (0.9%) as a standalone strategy, though an additional 8 studies (7.5%) combined osteoinductive and angiogenic cues. This reveals a striking imbalance: vascularization strategies are dramatically underutilized compared to osteogenic approaches.

**Table 6:** Bio-additives in craniomaxillofacial biofabrication.

Category	Bio-Additive	Primary Function	Application	Representative Articles
Growth Factors and Cytokines	BMP-2/BMP-7	Osteoinduction	Bone, Periodontal Complex	(32, 104)
	VEGF	Angiogenesis	Bone, Vascularized Constructs	(29)
	SDF-1	Stem Cell Homing	Bone, Periodontal Complex	(26, 98)
	CTGF/TGFβ3	Fibrocartilaginous Differentiation	TMJ Disc	(67)
Peptides and Signaling Molecules	RGD	Cell Adhesion, Osteogenic Differentiation	Bone	(37)
	KSL-W	Antimicrobial Activity	Bone (Infected Defects)	(41)
	BMP Peptide	Odontogenic Differentiation	Dentin-Pulp Complex	(81)
Small Molecules and Drugs	Strontium Ranelate	Osteogenesis, Osteoclast Inhibition	Bone	(47)
	Metformin	Osteogenesis, Multi-tissue Regeneration	Bone, Vasculature, Nerve	(91)
	Propolis	Antibacterial, Cell Proliferation	Bone	(33)
	Dipyridamole	Osteoinduction	Bone	(62)
	EPA/DHA	Angiogenesis, Collagen Production	Gingiva	(84)
	Boric Acid	Osteogenic Differentiation	Bone	(54)
Inorganic and Ceramic Additives	Nacre	Osteoinduction, Mineralization	Bone (Alveolar)	(16)
	Nanosilicate	Osteoinduction, Mineralization	Bone	(48)
	Ceria Nanoparticles	Osteo-immunomodulation	Multi-Tissue Constructs	(92)
	Sr-doped Ceramics	Dual-Action Osteogenesis	Bone, Periodontal Complex	(108, 109)
	Graphene Oxide	Osteogenic Differentiation	Bone	(50)
	Amorphous Mg Phosphate	Osteogenic Differentiation	Bone	(25)
	NanoMgO	Osteogenesis, Angiogenesis	Bone	(34)
	Laponite	Rheological Modification, Printability	Dental Pulp	(69)
	Magnetic Nanoparticles	Mechanotransduction	Periodontal Complex	(111)
Vesicles and Cell Products	MSC-derived Exosomes	Angiogenesis, Osteogenesis	Bone	(51)
	hPDLCs-sEVs	Periodontal Lineage Differentiation	Periodontal Complex	(115)
	haMVs and Collagen I	Pre-formed Vascular Network	Bone	(53)
Genetic Material	miR-148b	Regulation of Osteogenesis	Bone	(30)

BMP, bone morphogenetic protein; CTGF, connective tissue growth factor; DHA, docosahexaenoic acid; EPA, eicosapentaenoic acid; haMVs, human microvessels; hPDLCs-sEVs, small extracellular vesicles from human periodontal ligament cells; KSL-W, antimicrobial peptide (KSL-W sequence); miR, microRNA; Mg; magnesium; MgO, magnesium oxide; MSC, mesenchymal stromal cell; RGD, arginine-glycine-aspartate peptide; SDF-1, stromal cell-derived factor 1; Sr, strontium; TGFβ3, transforming growth factor beta 3; TMJ, temporomandibular joint; VEGF, vascular endothelial growth factor.

In non-bone applications, bio-additives were applied in a target-specific manner, including chondrogenic factors (CTGF/TGF-β3) for TMJ disc regeneration, antimicrobial peptides (e.g., KSL-W) for infected defects, and odontogenic inductors (e.g., BMP peptides, MTA) for dentin–pulp complex models. Small molecules (metformin, dipyridamole) and bioactive ions (strontium, magnesium) were also used for immunomodulatory and mineralization-related effects.

### 4.7 Cross-dimensional relationships

**Figure 3:**
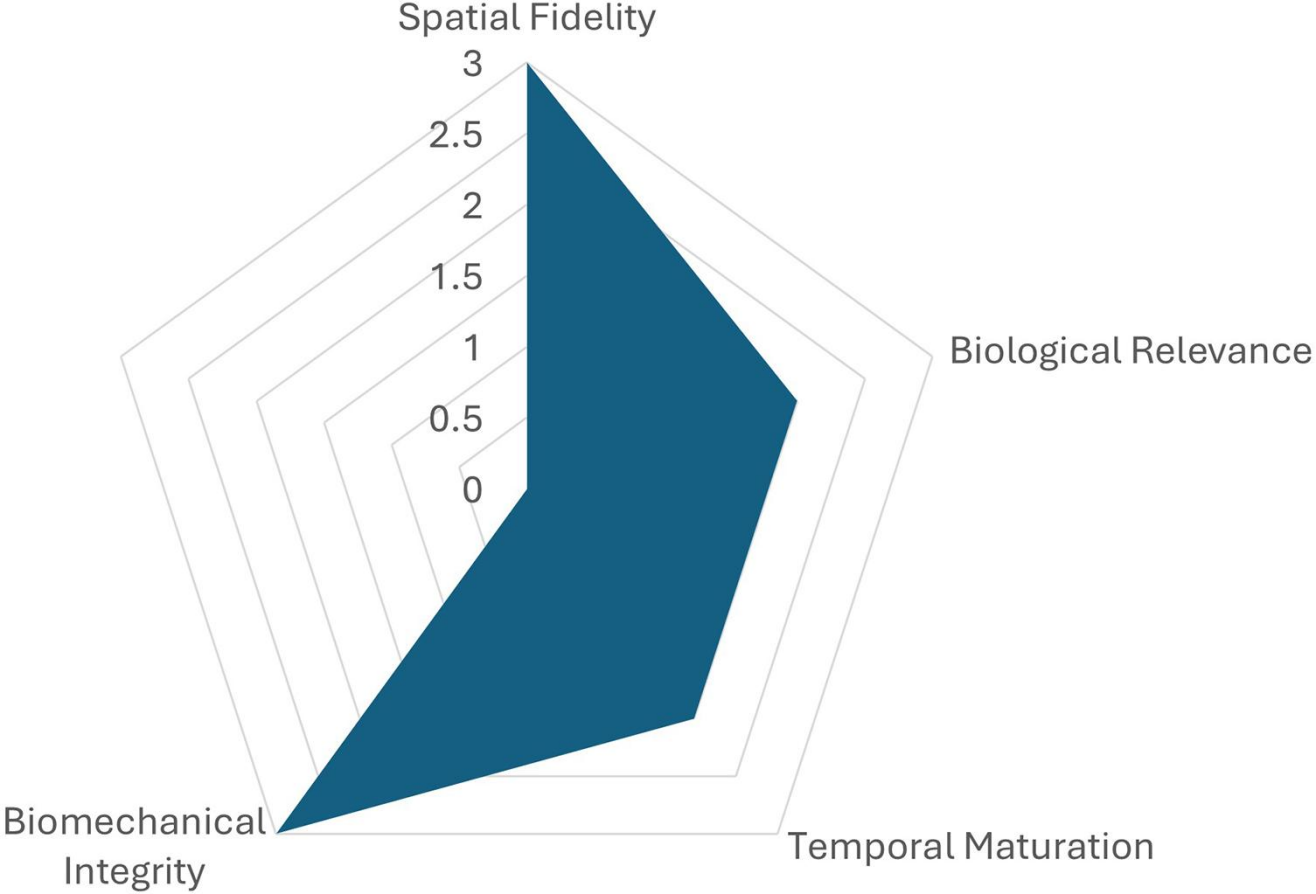
Complexity–validation matrix of CMF biofabrication studies. Heatmap showing study distribution across structural complexity (I–IV) and validation depth. Most approaches, even highly complex constructs, remain limited to *in vitro* and small-animal testing, with few advancing to large-animal studies and minimal proof-of-concept evidence, highlighting a persistent gap between engineering sophistication and clinically relevant validation.

Examination of construct complexity, fabrication approach, and translational stage revealed an uneven distribution of studies across validation levels (Figure 3). Even constructs designed to address higher structural complexity were most often evaluated only *in vitro* or in small-animal models, while relatively few progressed to large-animal testing or functional proof-of-concept. This suggests that advances in construct design have not been accompanied by equivalent progress in clinically relevant validation.

Supporting this observation, all whole-organ constructs (Level IV, *n* = 2) were tested in large animal models, whereas only 4 of 23 multi-tissue constructs (Level III, 17.4%) reached this stage. Mono-tissue (Level I) and dual-tissue (Level II) constructs were largely confined to *in vitro* or small-animal studies. Scaffold-assisted approaches accounted for 13 of the 17 large animal investigations (76.5%). Cell-laden strategies were used in 44 studies overall, but only 4 advanced to large animal testing. Hybrid and cell-free strategies were predominantly limited to early-stage research. Bio-additives were incorporated in 17 of 23 Level III multi-tissue constructs (73.9%), compared with 34 of 71 Level I mono-tissue constructs (47.9%).

## 5 Discussion

### 5.1 Interpreting the strategic map: convergence and the translational ambition gap

**Figure 4:**
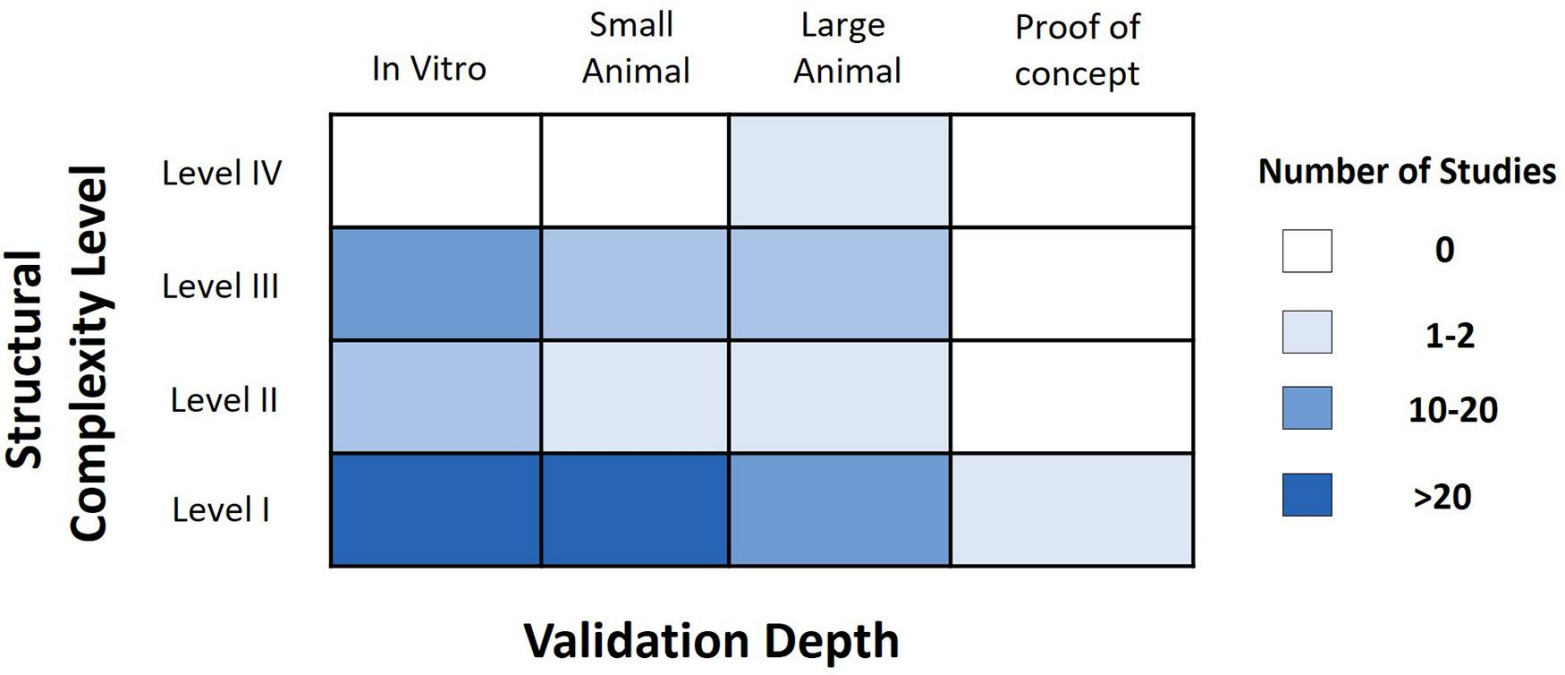
Four-domain analytical framework for evaluating translational readiness in CMF biofabrication. This conceptual framework, derived from the authors' synthesis of the literature, illustrates the relative emphasis observed across four key dimensions: spatial fidelity, biological relevance, temporal maturation, and biomechanical integrity. The uneven profile reflects the field's current focus on structural precision and mechanical stability, with comparatively less attention to long-term biological function and maturation—highlighting key barriers to clinically meaningful regeneration.

This scoping review maps the strategic contours of CMF biofabrication. Our synthesis of 107 studies reveals a field defined by strategic convergence and a measurable gap between engineering ambition and translational validation. To synthesize these multidimensional gaps, we propose a conceptual four-domain framework, derived from qualitative interpretation of the literature, encompassing spatial fidelity, biological relevance, temporal maturation, and biomechanical integrity. This framework is intended as an interpretative synthesis rather than a quantitative scoring or weighting system (Figure 4).

Geographically, research is concentrated, with China, the USA, and South Korea contributing 66.4% of studies, underscoring the resource-intensive nature of the field. Translational progression remains predominantly preclinical, with 89 of 107 studies (83.2%) conducted either *in vitro* or in small animal models. As noted by Varshney, Dwivedi (1), a significant portion of discoveries remains confined to laboratory stages, necessitating further validation. This early-stage skew is reflected in engineering complexity: the majority (66.4%) targeted mono-tissue regeneration (Level I), indicating a foundational focus. Bi-phasic (Level II) constructs were less common (10.3%), while multi-tissue (Level III) constructs represent a significant engineering challenge (21.5%). The most ambitious whole-organ (Level IV) constructs are exceedingly rare (1.9%). This distribution reveals a field cautiously advancing from simple to complex, with most efforts residing in a “comfort zone” of incremental, single-tissue validation—a pattern that fragments efforts and may constrain overall translational potential.

### 5.2 The dominant toolkit: technological conservatism as a structural bottleneck

Within this landscape, technological and material choices show remarkable uniformity, creating a dominant but potentially limiting paradigm. Our analysis quantifies a heavy reliance on extrusion-based bioprinting (EBB), employed in 68.2% of studies. While EBB is a versatile “workhorse,” its resolution—typically in the hundreds of microns—restricts reproduction of fine microstructures like capillary networks (4). This extrusion trade-off between practicality and precision is a central engineering constraint, as even advanced methods fall short of the ∼3 µm needed for capillaries (8). Material selection follows a similarly conservative trajectory. Composite systems, constituting 59.8% of studies, dominate by combining polymers like PCL with ceramics like HA. This reliance on a familiar toolkit, despite known challenges in achieving tissue-specific performance and batch-to-batch consistency, suggests material stagnation (119). The field faces a fundamental dilemma: balancing the stiffness needed for structure with the softness required for cell viability remains an unsolved, foundational challenge (2, 120). Consequently, materials are often chosen for printability or mechanical properties rather than their capacity to actively guide cell behavior through adhesion and signaling—a limitation common to many inert composite systems.

### 5.3 The translational disconnect: validating structure while neglecting function

A critical disconnect emerges when cross-analyzing ambition, model selection, and outcome reporting—the core of the translational chasm.

While the field designs increasingly complex multi-tissue constructs, our cross-dimensional analysis reveals that 19 of 23 Level III constructs (82.6%) were investigated exclusively *in vitro* or in small animal models. These models cannot replicate the biomechanical loads or scale of human CMF defects. As Mohd, Razali (5) caution, results from small animals should be interpreted carefully for human applications. For bone, studies overwhelmingly reported radiographic and histological evidence of formation, while quantitative assessments of mechanical integrity were far less common. For soft tissues, functional metrics like ligament biomechanics or sensory re-innervation were absent. A key mechanistic factor is the systematic neglect of the mechanical environment: most constructs are tested under static or low-load conditions, failing to expose cells to the mechanobiological cues critical for functional tissue maturation and integration. Consequently, the field demonstrates structural feasibility but provides limited evidence of functional efficacy in physiologically relevant contexts, reinforcing a persistent “preclinical–clinical chasm” (1).

### 5.4 Quantifying biological blind spots: from vascularization to regulation

Beyond validation models, our data quantify specific biological and strategic gaps underpinning the translational disconnect. A fundamental biological limitation is the deficit in coupled signaling. Osteogenic programs are often initiated without coordinated angiogenic induction. While osteoinduction is prioritized (51% of bone studies), only 7.5% of all studies incorporated angiogenic factors. This uncoupled approach compromises cell survival in thick constructs and risks central necrosis (5, 8). These biological limitations are further worsened by inconsistencies in reporting, variability in polymer batches, and limited long-term safety data. Collectively, these factors lead to a reproducibility crisis (119).

Together, these limitations culminate in a regulatory and commercial impasse. Bioprinted constructs remain in a regulatory gray zone, with no FDA-approved tissues and few registered clinical trials to date (2, 4). High costs, extended cell expansion protocols, and unproven superiority over the autograft gold standard continue to restrict clinical translation (8, 119).

### 5.5 A strategic roadmap- toward computationally enabled, functional translation

Bridging the identified chasm requires a paradigm shift from iterative optimization to intelligent, function-driven biofabrication. We propose four integrated pillars:

- Hybrid biofabrication: Move beyond extrusion dependence. Hybrid fabrication, combining EBB with high-resolution (e.g., DLP) techniques, is essential to create hierarchical vasculature (121).
- Functional benchmarking: Funding should prioritize large-animal studies for complex constructs and mandate standardized functional outcomes (5, 119). This addresses the animal model fidelity gap.
- Predictive design: Leverage AI and computational modeling to transition from trial-and-error. Machine learning can guide bioink design and predict optimal bio-additive cocktails, while “digital twin” simulations can de-risk performance (120).
- Dynamic biomaterials: Advance beyond static composites. The field must develop bioactive material systems that provide spatiotemporal cues for vascularization, immunomodulation, and coupled tissue maturation. The proactive co-development of agile regulatory pathways with governing bodies is the essential step to translate these pillars into clinically viable, personalized manufacturing frameworks (4).

## 6 Limitations

This review is constrained by its inclusion of English-language publications and a defined timeframe. As a scoping review, it maps evidence characteristics but does not formally appraise study quality.

## 7 Conclusion

The CMF field, serving as a demanding model system, reveals key transferable challenges for biofabrication: materials must be biologically instructive to actively guide cell behavior, vascular and tissue-specific signaling must be temporally coordinated, and functional outcomes must be validated in physiologically relevant environments. While CMF anatomy presents unique complexities, the strategic and mechanistic insights derived here—from hybrid fabrication approaches to function-driven validation—offer a practical roadmap for regenerating any complex, load-bearing tissue composite, directly informing the pathway to clinical translation.

## Data Availability

The original contributions presented in the study are included in the article/Supplementary Material, further inquiries can be directed to the corresponding author.
